# Open Thoracoabdominal Aortic Replacement in a Long-Term Survivor of Early-Onset Marfan Syndrome

**DOI:** 10.70352/scrj.cr.26-0130

**Published:** 2026-06-09

**Authors:** Yuma Yokoyama, Tsutomu Ito, Yorihiko Matsumoto, Hideyuki Shimizu

**Affiliations:** 1 Department of Cardiovascular Surgery, Keio University, Tokyo, Japan; 2 Department of Cardiovascular Surgery, Tokyo Dental College Ichikawa General Hospital, Ichikawa, Chiba, Japan

**Keywords:** early-onset Marfan syndrome, dissecting aortic aneurysm, thoracoabdominal aortic replacement

## Abstract

**INTRODUCTION:**

Early-onset Marfan syndrome (eoMFS) is a rare and severe connective tissue disorder marked by cardiovascular complications in early life. It carries a poor prognosis, often causing death within the first 2 years of life due to heart failure. However, aggressive medical management and early surgical intervention can result in long-term survival, and these patients may subsequently develop aortic disease in early adulthood. Herein, we present a rare case in which we successfully treated an aortic dissection in a patient with eoMFS.

**CASE PRESENTATION:**

A 19-year-old man with eoMFS developed a complicated Stanford type B aortic dissection with bilateral lower-limb malperfusion. He was diagnosed with eoMFS in early childhood. He underwent mitral and tricuspid valve repair at 18 months of age, followed by 2 additional mitral valve repairs at ages 3 and 7 for recurrent regurgitation, which enabled stable management of heart failure. In addition, aortic root replacement for annuloaortic ectasia was performed at age 11. After the diagnosis of complicated aortic dissection at age 19, thoracic endovascular aortic repair was performed to restore distal perfusion. Close follow-up revealed rapid aneurysmal dilation, and definitive open thoracoabdominal aortic replacement (TAAR) was planned. Surgical challenges included prior thoracic surgeries, difficulty in surgical exposure of the distal arch, and the inability to place a cerebrospinal fluid drain due to severe scoliosis. A straight incision with a rib-cross thoracotomy approach was used for optimal access. The aorta was reconstructed under deep hypothermia with selective visceral perfusion. Postoperatively, the patient required prolonged respiratory support due to chest deformity and pneumonia, but was eventually discharged without cerebral infarction or spinal cord ischemia.

**CONCLUSIONS:**

eoMFS is an extremely rare disease with a high mortality rate; however, survival beyond infancy may be achievable with appropriate valve interventions. These patients may develop aortic disease in early adulthood, and critical technical considerations are required for open TAAR, taking into account the altered anatomy and prior interventions.

## Abbreviations

CSF
: cerebrospinal fluid
eoMFS
: early-onset Marfan syndrome
MFS
: Marfan syndrome
SIRC
: straight incision with rib-cross thoracotomy
TAAR
: thoracoabdominal aortic replacement
TEVAR
: thoracic endovascular aortic repair

## INTRODUCTION

eoMFS is a rare and severe form of MFS characterized by cardiovascular complications in early life. This form has a significantly poorer prognosis than classic MFS. Many patients with eoMFS die within the first 2 years of life, most commonly from congestive heart failure, whereas patients with classic MFS most often die from aortic dissection or rupture, typically later in life.^1)^ Although early mortality in eoMFS remains high, advances in surgical management have made survival beyond infancy achievable.^1)^ As more patients reach adolescence and adulthood, long-term outcomes will depend not only on early valvular interventions but also on how we address late-onset aortic complications. We present a rare case of a patient with eoMFS who developed a complicated Stanford type B aortic dissection in early adulthood, which was successfully treated with open TAAR.

## CASE PRESENTATION

The patient was one of a pair of monozygotic twins and exhibited dolichocephaly, scoliosis, and arachnodactyly. Initial echocardiography showed mitral valve regurgitation and a dilated aortic root. Genetic testing confirmed a missense mutation in exon 26 of *FBN1*, consistent with the eoMFS phenotype. This patient was previously reported as Patient 1 by Maeda et al.,^2)^ where the detailed genetic findings are provided. His monozygotic twin brother, who also exhibited a Marfanoid habitus, died suddenly at 4 months of age; no cause of death was identified. At 18 months, he underwent mitral and tricuspid valve repair for severe regurgitation. Recurrent mitral regurgitation necessitated 2 further mitral valve repairs at ages 3 and 7, as reported by Kitahara et al.^3)^ We performed a modified Bentall procedure for annuloaortic ectasia and severe aortic insufficiency at age 11, using an On-X Aortic Heart Valve (23 mm; CryoLife, Kennesaw, GA, USA) and a Gelweave Valsalva graft (26 mm; Vascutek, Inchinnan, Scotland, UK). He has achieved long-term survival without recurrence of heart failure after these early interventions.

**Fig. 1:**
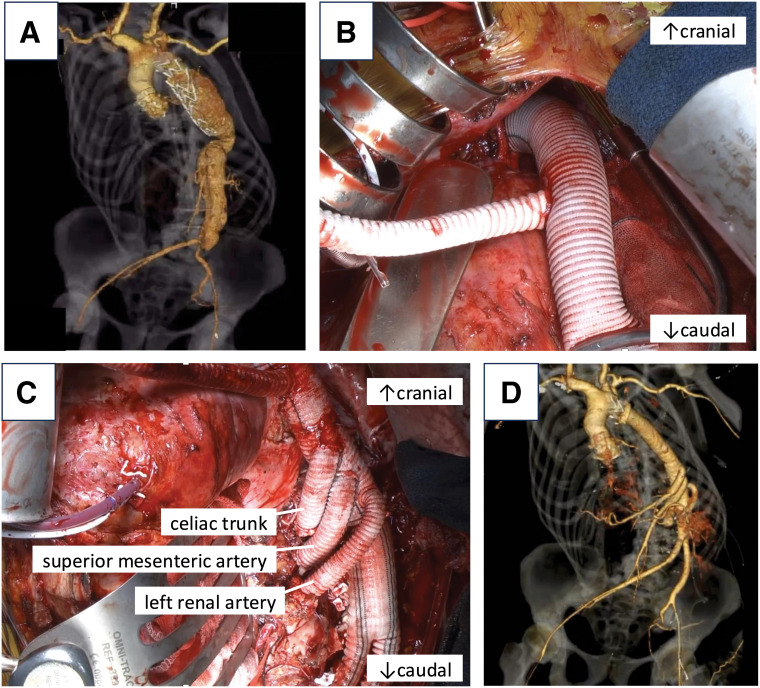
Preoperative imaging, key intraoperative steps, and postoperative outcomes. (**A**) Preoperative CT angiography showing progressive aneurysmal dilation of the stented aorta. (**B**) Intraoperative view of the proximal graft anastomosis via SIRC. (**C**) Reimplantation of the celiac, superior mesenteric, and bilateral renal arteries into the graft. (**D**) Postoperative CT angiography showing no abnormal findings. SIRC, straight incision with rib-cross thoracotomy

At age 19, he was found to have a Stanford type B aortic dissection complicated by malperfusion of both lower extremities. We performed emergent TEVAR using a distal stent graft (Zenith Alpha, 26 × 104 mm; Cook Medical, Bloomington, IN, USA) and a proximal stent graft (Zenith Alpha, 30 × 109 mm), which successfully reestablished distal perfusion. Post-TEVAR CT demonstrated that the aortic diameters increased over approximately 5 months as follows: distal aortic arch, from 34.8 to 50.0 mm; descending thoracic aorta, from 33.5 to 47.6 mm; abdominal aorta at the level of the celiac artery, from 30.0 to 37.6 mm; and distal abdominal aorta, from 16.1 to 26.1 mm (**Fig. 1A**). Given this rapid post-TEVAR aortic expansion, a definitive open TAAR was undertaken approximately 8 months after TEVAR. Pre-operative imaging failed to identify the artery of Adamkiewicz because spinal-correction rods caused artifacts, and the marked scoliosis also precluded placement of a CSF drainage catheter.

Given the small caliber of the femoral artery, it was considered unsuitable for arterial inflow; therefore, the left common iliac artery and the left axillary artery were used instead. Venous drainage was established via the right atrium through the right femoral vein, and an additional pulmonary artery vent and a left ventricular apical vent were placed to prevent distension, thereby establishing cardiopulmonary bypass.

The descending thoracic aorta was exposed through an SIRC. The aorta was reconstructed using sequential segmental cross-clamping to minimize spinal cord ischemia. The descending aorta was initially clamped at its mid-portion. An open proximal anastomosis using a 4-branched vascular graft (Gelweave Coselli Thoracoabdominal Graft, 20 mm; Vascutek) was performed just distal to the left subclavian artery under hypothermic circulatory arrest of the upper body (**Fig. 1B**). The previously implanted stent graft was transected, leaving 1 bare stent. After de-airing via low-flow retrograde perfusion through the right atrial cannula, upper-body perfusion through the graft side branch was initiated. The nadir bladder temperature was 19.1°C, and the duration of circulatory arrest was 42 min.

Although back-bleeding was observed from several small intercostal arteries, no intercostal arteries of sufficient caliber to warrant reimplantation were identified. A bifurcated vascular graft (Gelsoft Plus Bifurcate, 18 × 9 mm) was anastomosed to both common iliac arteries while the visceral branches were selectively perfused. Subsequently, the bifurcated graft was connected to the 4-branched graft. Finally, the visceral branches were sequentially reconstructed to the 4-branched graft (**Fig. 1C**). The cardiopulmonary bypass time was 327 min, and the total operative time was 896 min.

Postoperative CT confirmed patency of all visceral branches (**Fig. 1D**). During the postoperative course, he required intensive care for restrictive ventilatory impairment and pneumonia, necessitating a temporary tracheostomy. However, no cerebral infarction or spinal cord ischemia occurred postoperatively. He was successfully weaned from mechanical ventilation and was discharged home on POD 94.

## DISCUSSION

There is currently no universally accepted definition of eoMFS. Recently, Zarate et al. proposed a clinical scoring system for eoMFS that demonstrated excellent diagnostic performance, with a cutoff value of 16 out of a maximum score of 38.^4)^ When this scoring system was applied to the present case, the total score was 25, supporting the diagnosis of eoMFS.

In our case, emergent TEVAR was initially performed for acute Stanford type B aortic dissection with extensive lower-limb ischemia to restore distal perfusion. Although TEVAR should generally be avoided in patients with MFS, it should remain a therapeutic option in life-threatening situations requiring urgent restoration of perfusion, even in patients with underlying connective tissue disorders. We acknowledge that aneurysmal enlargement may progress more rapidly than usual after TEVAR in such patients. Therefore, close follow-up with frequent CT imaging is essential, and the timing of definitive surgical repair with graft replacement should be carefully determined.

When TAAR is required in an adult survivor of eoMFS, several technical challenges arise.

First, multiple previous surgical procedures and inflammation from aortic dissection can cause the lungs to become densely adherent to the pleura. In this case, the left lung was densely adherent to the pericardium, diaphragm, and chest wall. Meticulous dissection was performed, and the partially injured visceral pleura was repaired with sutures. Because severe scoliosis imposes a restrictive ventilatory defect, careful attention should be paid to dissection of adhesions around the lung and to preservation of the phrenic nerve to prevent postoperative respiratory complications.

Second, pronounced spinal curvature can hinder exposure of the distal arch for the proximal anastomosis. In this case, we utilized the SIRC approach, which is an exposure technique for an extensive aortic aneurysm,^5)^ to enhance access to the aortic arch. By using this technique, the dissected aortic wall was removed entirely and the proximal anastomosis was safely accomplished.

Third, severe spinal deformity may preclude lumbar CSF drainage. To prevent spinal cord ischemia, careful attention was paid intraoperatively and postoperatively to avoid excessive decreases in mean arterial pressure and hemoglobin levels. In addition, we carried out the repair under deep hypothermia, which may offer a protective effect on the spinal cord compared with arch clamping, as reported by Yoo et al.^6)^ Although the intercostal arteries were not reconstructed, no postoperative paraplegia was observed, suggesting that spinal perfusion was maintained by the extensive collateral network supplied by the subclavian and internal iliac arteries. This is referred to as the “collateral-network concept,” recently reviewed by Ohira et al.^7)^ Emergent TEVAR performed before TAAR may have acted as ischemic preconditioning, stimulating enlargement of the collateral network.

## CONCLUSIONS

We experienced a rare case of eoMFS in which the patient developed aortic dissection in early adulthood and was successfully treated surgically. Although appropriate valvular interventions may enable survival beyond infancy in patients with eoMFS, these patients remain at risk of developing aortic disease in early adulthood. Aortic disease in such patients requires careful surgical planning that fully takes into account prior treatment history and physical characteristics.
